# Supplementary material for: Resveratrol for the Management of Human Health: How Far Have We Come? A Systematic Review of Resveratrol Clinical Trials to Highlight Gaps and Opportunities
Source: Int J Mol Sci. 2024 Jan 6;25(2):747. doi: 10.3390/ijms25020747 (PMC10815776; doi:10.3390/ijms25020747)
Supplement: Supplementary file 1 [file ijms-25-00747-s001.zip › ijms-2780248-supplementary.pdf]

Supplementary Materials

Methods

Search Strategies

All searches were run on 24th February 2023, from database inception.

Ovid MEDLINE(R) ALL <1946 to February 23, 2023>

1 Resveratrol/ 10582

2 resveratrol*.tw. 16011

3 Resvida.tw. 9

4 Longevinex.tw. 13

5 RESV.tw. 201

6 "SRT-501".tw. 13

7 SRT 501.tw. 1

8 SRT501.tw. 13

9 "trans-resveratrol".tw. 19

10 "3,4',5 stilbenetriol".tw. 1

11 "3,4',5 trihydroxystilbene".tw. 225

12 "5 (4 hydroxystyryl)benzene 1,3 diol".tw. 0

13 "3,5,4'-Trihydroxystilbene".tw. 258

14 "cis-Resveratrol".tw. 122

15 1 or 2 or 3 or 4 or 5 or 6 or 7 or 8 or 9 or 10 or 11 or 12 or 13 or 14 16458

16 exp animals/ not humans.sh. 5095326

17 15 not 16 11721

18 (dose* or dosage* or dosing or regimen* or mg or 250mg or 500mg or mcg or milligram* or 250milligram* or 500milligram* or microgram*).mp. 3851603

19 exp drug administration routes/ or exp drug administration schedule/ or drug dosage calculations/ 729418

20 18 or 19 4017067

21 17 and 20 3396

Embase <1974 to 2023 February 23>

1	resveratrol/	28301
2	resveratrol*.tw.	20697
3	Resvida.tw.	29
4	Longevinex.tw.	22
5	RESV.tw.	295
6	"SRT-501".tw.	20
7	SRT 501.tw.	68
8	SRT501.tw.	20
9	"trans-resveratrol".tw.	62
10	"3,4',5 stilbenetriol".tw.	1
11	"3,4',5 trihydroxystilbene".tw.	273
12	"5 (4 hydroxystyryl)benzene 1,3 diol".tw.	0
13	"3,5,4'-Trihydroxystilbene".tw.	318
14	"cis-Resveratrol".tw.	139
15	1 or 2 or 3 or 4 or 5 or 6 or 7 or 8 or 9 or 10 or 11 or 12 or 13 or 14	30205
16	exp experimental organism/	853396
17	animal tissue/	1627251
18	animal cell/	1746641
19	exp animal disease/	299594
20	exp carnivore disease/	50398
21	exp bird/	261911
22	exp experimental animal welfare/	3340
23	exp animal husbandry/	62144
24	animal behavior/	90235
25	exp animal cell culture/	13053
26	exp mammalian disease/	183814
27	exp mammal/	29087932
28	exp marine species/	8965
29	nonhuman/	7382720
30	animal.hw.	6056672

31 16 or 17 or 18 or 19 or 20 or 21 or 22 or 23 or 24 or 25 or 26 or 27 or 28 or 29 or 30 32324945

32 31 not human/ 7319952

33 15 not 32 20783

34 (dose* or dosage* or dosing or regimen* or mg or mcg or milligram* or microgram*).mp. 4430916

35 exp dose/ 826840

36 exp drug administration/ 1263095

37 34 or 35 or 36 5257153

38 33 and 37 4497

Cochrane Database of Systematic Reviews

Cochrane Central Register of Controlled Trials (CENTRAL)

Date Run: 24/02/2023 14:31:34

ID Search Hits

#1 MeSH descriptor: [Resveratrol] this term only 357

#2 resveratrol* 755

#3 Resvida 13

#4 Longevinex 3

#5 RESV 5

#6 "SRT-501" 1

#7 SRT 501 6

#8 SRT501 3

#9 "trans-resveratrol" 94

#10 "3,4',5 stilbenetriol" 0

#11 "3,4',5 trihydroxystilbene" 2

#12 "5 (4 hydroxystyryl)benzene 1,3 diol" 0

#13 "3,5,4'-Trihydroxystilbene" 5

#14 "cis-Resveratrol" 1

#15 {OR #1-#14} 760 (13 Reviews, 744 Trials included; 2 Protocols, 1 Clinical Answer not included).

Clinicaltrials.gov strategy

In intervention/treatment field:

resveratrol* OR Resvida OR Longevinex OR RESV OR “SRT-501” OR “SRT 501” OR SRT501 OR "trans-resveratrol" OR "3,4',5 stilbenetriol" OR "3,4',5 trihydroxystilbene" OR "3,5,4'-Trihydroxystilbene" OR "cis-Resveratrol"

All studies included

185 results

Supplementary Table S1. Summary of all non-randomised controlled trials (non-RCTs) identified through the search strategy that involved administration of resveratrol (RSV) in a purified form. Also listed under each numbered primary RCT publication are any associated studies that used samples and/or data from the original trial. These linked studies are shown with the same background colour (white or grey) and are separated by dashed lines.

	**Study** (trial registration identification)	**Type of trial**	**Primary aim or outcome**	**Participants**	**Dose and schedule**	**Number of participants**	**Adverse Events**	**Main findings**
**1**	Alonso 2017[170]	PK study	Skin penetration of RSV	Healthy female volunteers	5% RSV (w/v) in ethanol/water (70:30 v/v). Topical formulation. Single application of 500 mg/cm^2	6	Not stated	High RSV throughout stratum corneum surface layers, but lower amounts in upper layers of epidermis. RSV maintained antiradical activity following topical application, with antioxidant efficacy higher in inner layers of the stratum corneum.
**2**	Amiot 2013[171]	PK study	PK, bioavailability, toxicity	Healthy volunteers	40 mg given as a single dose for both formulations	15 on powder RSV formulation; 15 on a galenic RSV formulation	Zero reported	Soluble galenic t-RSV was well absorbed, giving 0.1-6.0 mM post-dose for several hours, despite being metabolised into glucuronide and sulfate conjugates. Blood levels were negligible when administered as a dry powder. The galenic formulation gave 8.8-fold higher t-RSV plasma levels vs RSV powder.
**3**	Andrade 2019[172]	Placebo controlled pilot study	Expression of FNDC5 in adipose tissue	Healthy obese volunteers	500 mg once daily or placebo for 4 weeks	10 on RSV; 10 on placebo	Not stated	Increased FNDC5 expression was observed in mouse and human subcutaneous adipose tissue.

**4**	Bailey 2021[173]	Double blind randomised trial (not placebo controlled)	Characterisation of single-dose RSV PK with or without piperine.	Healthy participants	Single dose of 2.5 g RSV alone or with piperine at 5 mg or 25 mg	8 per group (3 groups); all had resveratrol	17 (71%) of participants had at least one AE; 10 (42%) had multiple AEs. RSV was tolerated without significant sequelae, with no significant changes in lab safety values within patients/ across treatment groups 24 h post-dose. Grade 1-2 toxicity was observed in 50-75% participants across all treatment groups. Transient grade 1 nausea 24 h post-dose was the most common AE reported by 1-2 participants in each group, followed by grade 1 headache.	Coadministration of resveratrol with piperine at 5 and 25 mg did not sufficiently alter RSV or RSV glucuronide PK as observed in murine modelling.
**5**	Bedada 2015[154]	Non-blinded, open-label, two-period, sequential study	Effect of RSV pre-treatment on metabolism and PK of carbamazepine (CBZ); Evaluate effect of RSV on CYP3A4 enzyme activity	Healthy volunteers	500 mg RSV once daily for 10 days; with a single dose of 200 mg CBZ alone or in combination with RSV	12	Zero reported	RSV pre-treatment enhanced bioavailability of CBZ. RSV altered CYP3A4 enzyme activity and PK of CBZ (may be attributed to CYP3A4 inhibition).

**6**	Bedada 2016[153]	Crossover	Effect of RSV treatment on CYP2E1 mediated metabolism	Healthy volunteers	RSV 500 mg once daily for 10 days. Chlorzoxazone (CHZ) 250 mg taken alone or in combination with RSV as a single dose	12	Zero reported	RSV pre-treatment significantly enhanced C_{max}, AUC, $T_{1/2}$ and significantly decreased elimination rate constant, apparent oral clearance and apparent volume of distribution of CHZ vs control. Altered CHZ PK might be attributed to RSV-mediated CYP2E1 inhibition.
**7**	Bedada 2016[174]	Crossover	Effect of RSV treatment on metabolism and PK of diclofenac.	Healthy volunteers	RSV 500 mg once daily for 10 days. Diclofenac (100 mg) taken alone or in combination with RSV as a single dose	12	Zero reported	RSV treatment significantly enhanced C_{max}, AUC and $T_{1/2}$ of diclofenac and significantly decreased the elimination rate constant, apparent oral clearance of diclofenac vs control.
**8**	Blanchard 2014[175]	PK study	C_{max} of RSV following administration of RSV lozenge	Healthy volunteers	One lozenge containing 140 mg RSV given as a single dose	2	Zero reported	RSV-ribose matrix lozenge achieved greater C_{max} and entered the bloodstream faster than previously reported dosage forms for gastrointestinal absorption
**9**	Bode 2013 (DRKS00004311)[176]	PK/ metabolism study	Identify new metabolites; elucidate interindividual routes of RSV conversion by the human gut microbiota; identify the bacterial species involved	Healthy volunteers	0.5 mg RSV/Kg body weight in a colloid formation on a single occasion	12	Not stated	Dihydroresveratrol and 2 previously unknown bacterial t-RSV metabolites were identified in vitro and *in vivo*: 3,4'- dihydroxy-*trans*-stilbene and 3,4'-dihydroxybibenzyl (lunularin). Two strains, Slackia equolifaciens and Adlercreutzia equolifaciens, were identified as dihydroresveratrol producers.

**10**	Boocock 2007[156]	PK study	Safety and PK	Healthy volunteers	0.5, 1, 2.5 or 5 g RSV as a single oral dose	10 per dose group (40 in total). No untreated control comparator	51 (in 23 volunteers) deemed unlikely to be drug related side effects: Raised urea; raised creatinine; low total protein; raised phosphate; low phosphate; raised bilirubin; raised calcium; raised glucose; low CO_2; raised lactate dehydrogenase; low lactate dehydrogenase; raised cholesterol; low WBC count; raised neutrophil count; raised lymphocyte count; raised eosinophil count; two individuals on 1.0 g RSV experienced an increase in blood bilirubin to 24Amol/L, and an increase in alanine aminotransferase to 55 IU/L on day 4 post-dose, resolving in the subsequent week. Low basophil count; raised platelet count; low hematocrit; raised	RSV did not cause serious adverse events. RSV and six metabolites were recovered from plasma and urine. Peak plasma levels of RSV at the highest dose were 539 +/- 384 ng/mL (2.4 Mmol/L, mean +/- SD; n = 10), which occurred 1.5 h post-dose. Peak levels of two monoglucuronides and resveratrol-3-sulfate were 3- to 8-fold higher. AUC plasma values for resveratrol-3-sulfate and resveratrol monoglucuronides were up to 23 times greater than those of resveratrol.

							chloride; cellulitic foot infection; flatulence; nausea; loose stool; panic attack; headache. Possibly drug related side effects: raised bilirubin; raised alanine aminotransferase.	

	Boocock 2007 [177]		Develop analytical methodology combining separation of major RSV metabolites and allowing quantitation of parent compound in a short run time					A rapid, sensitive and accurate method for analysis of RSV and its metabolites in human plasma and urine for PK studies was developed.
**11**	Briskey 2020 (ACTRN12619000953134)[158]	PK study	Compare PK of a single dose of commercially available RSV(Veri-te) with a resveratrol-LipiSpers delivery complex (Veri-Spers)	Healthy adults	A single dose of 150 mg of Veri-te RSV or Veri-te RSV with LipiSperse at a dose of 75 mg or 150 mg	12 participants on 150 mg of RSV; 12 on 75 mg RSV with LipiSperse; 13 on 150 mg RSV with LipiSperse	Zero reported	150 mg of Veri-Sperse® had a 2-fold increase in absorption (AUC) and a 3-fold increase in C_{max} of *trans*-RSV conjugates compared to 150 mg Veri-te. There was no statistical difference between 75 Veri-Sperse and 150 mg Veri-te for AUC or C_{max} of RSV conjugates
**12**	Brotons-Canto 2020 [159]	PK study	Assessing impact of the nano-encapsulation of RSV on its human PK.	Healthy volunteers	RSV (250mg) loaded in zein nanoparticles and given as a single dose as an aqueous suspension	16	Zero reported	Formulation was well tolerated and showed quantifiable plasma levels of RSV and its metabolites
**13**	Brown 2010 (NCT 00098969)[146]	Phase 1 safety and PK study	Safety & PK of parent RSV and its major metabolites; Effects on circulating levels of IGF-1 and IGFBP-3	Healthy volunteers	0.5, 1, 2.5 or 5 g RSV once daily for 29 days	10 per dose group (40 in total). No untreated control as comparator	42 AEs in 28 patients. RSV at 2.5 and 5 g caused mild to moderate gastrointestinal symptoms. Raised blood bilirubin; skin discoloration; cystitis; abdominal pain; acne; cramp; diarrhoea;	RSV was safe, but the 2.5 and 5 g doses caused mild to moderate gastrointestinal symptoms. Resveratrol-3-*O*-sulfate, resveratrol-4'-*O*-glucuronide and resveratrol-3-*O*-glucuronide were major plasma metabolites. RSV caused a decrease in circulating IGF-1 and IGFBP-3 compared to pre-dosing values.

							discomfort on passing faeces; fatigue; flatulence; nausea; pruritis; chest pain; dizziness; dry mouth; red/itchy eyes; urine colour change	
**14**	Cai 2015[157]	PK, tissue distribution study	Compare plasma and colorectal tissue distribution and activity of RSV at a low dietarily-relevant dose vs an intake 200 times higher	Healthy volunteers and colorectal cancer patients (two studies)	RSV 5 mg or 1 g daily as a single dose in healthy volunteers, and for 7 days in colorectal cancer patients	10 per dose group (20 healthy volunteers; 20 colorectal cancer patients).	Not stated	Peak plasma concentrations of 0.6 and 137 mM for intakes of 5 mg and 1 g, respectively
**15**	Cai 2021 (EudraCT 2007-002131-91)[178]	PK, tissue distribution study	Compare concentrations of RSV and metabolites in prostate tissue after ingestion of a pharmacological and dietary RSV dose	Patients with suspected prostate cancer	RSV 5 mg or 1 g daily for 7-14 days. Control patients were untreated.	10 on 5 mg RSV; 10 on 1 g RSV; 10 untreated control patients	2 reported as being possibly caused by RSV: diarrhoea, abdominal pain (which were each experienced by a single patient on the 1 g dose) AE grade 1	[^{14}C]-Resveratrol species were detectable in prostate tissue of all patients analysed, but were lower than we previously reported in plasma and colorectum after identical doses.

**16**	Chekalina 2016[179]	Non-randomised study	Effect of RSV vs quercetin on endothelial dysfunction, systemic inflammation, blood lipid profile and hemocoagulation in patients with stable coronary artery disease (CAD)	Patients with CAD: stable angina pectoris, FC II, CH 0-I; Healthy volunteers as controls	100 mg RSV daily for 2 months	30 on RSV plus standard care; 33 controls on standard care alone; 30 on quercetin (3 g per day) plus standard care.	Not stated	RSV significantly reduced TNF-α and the number of endothelial microparticles in peripheral blood. No changes to blood lipids observed.
**17**	Chow 2010[151]	Single arm pilot study	Effect on drug and carcinogen metabolising enzymes	Healthy volunteers	1 g RSV once daily for 4 weeks	42 on RSV	21 (across 42 people - possibly/probably related). Diarrhoea; heartburn; increased appetite; mood alteration; menstrual changes; vivid dreams; hot flashes; insomnia; decreased appetite; flatulence; nausea; abdominal pain; urine odour	RSV inhibited phenotypic indices of CYP3A4, 2D6, and 2C9, and induced phenotypic index of 1A2

**18**	Chow 2014 (NCT370889)[180]	Single arm pilot study	Change in serum estradiol levels	Healthy post-menopausal women	1 g RSV once daily for 12 weeks	40 (no control untreated comparator)	142 (including non RSV-related). One participant had asymptomatic Grade 4 elevation of liver enzymes at end of study intervention. Two subjects had Grade 3 skin rashes. The remaining AEs were Grade 1 or 2 events. Most common AEs were diarrhoea and increased total cholesterol, reported in 30% and 27.5% of the subjects, respectively. Other AEs were classed as blood disorders; ear disorders; eye disorders; GI disorders; immune system disorders; infections; metabolism; musculoskeletal; neoplasms; nervous system; psychiatric; renal; reproductive; respiratory; skin; vascular.	RSV did not significantly change serum concentrations of estradiol, estrone, and testosterone but did lead to an average of 10% increase in the concentrations of sex steroid hormone binding globulin (SHBG). RSV intervention resulted in an average of 73% increase in urinary 2-hydroxyestrone (2-OHE1) levels leading to a favourable change in urinary 2-OHE1/16α-OHE1 ratio

**19**	Crandall 2012[181]	Dose-escalating pilot study	Improvement to impaired glucose tolerance	Adults aged 65 and older with impaired glucose tolerance (IGT)	RSV 1, 1.5 or 2 g once daily for 4 weeks	10 in total (but not clear how many on each dose). No control untreated comparator.	3 AEs possibly related to RSV: Diarrhoea; Hot flashes. No SAEs.	RSV improves insulin sensitivity and post-meal plasma glucose in subjects with IGT at 1 and 2 g/day.
**20**	DeGroote 2012[182]	Pilot study	Effect of Resveratrol triphosphate (RTP) vs RSV vs catechin-rich grape seed extract (CGSE) on blood parameters related to oxidative stress in obese subjects.	Healthy obese adults	28 days of placebo followed by 28 days of equimolar daily doses (0.66 mmol) of either RSV, RTP or CGSE. Daily amount of RSV corresponds to 150 mg	10 on RSV; 12 on RTP;10 on CGSE	Zero reported	RTP and CGSE showed better antioxidant activities than RSV.
**21**	Diaz 2019[183]	Placebo controlled single blind crossover	Improvement to flow mediated dilation (FMD) and VO_2	Patients with history of coronary artery disease	Crossover design. RSV or placebo (330 mg) every 8 h for 3 days then one dose in the morning of 4th day; wash out of 7 days.	10	Not stated	Acute high dose supplementation with RSV improved FMD in patients after coronary artery bypass grafting surgery but impaired FMD in patients who underwent percutaneous coronary intervention.

**22**	Fabbrocini 2011[184]	Single blind, self-controlled pilot study	Effect of topical RSV on acne vulgaris	Patients with facial acne vulgaris	RSV in carboxymethyl-cellulose-based gel at 0.001% w/w (1 mg/g) applied once daily to right side of face for 60 days. Hydrogel vehicle applied to left side of the face.	20	Not stated	53.75% mean reduction in the GAGS score on RSV-treated sides of face vs 6.10% on vehicle-treated side of face. Histology showed 67% mean reduction in average area of microcomedones on RSV-treated side of the face.
**23**	Gualdoni 2014[185]	Randomised open label pilot study	Cytokine levels	Healthy male subjects	5 g of RSV or placebo taken as a single dose	8 on RSV; 2 on placebo	Zero reported	RSV significantly increased tumour necrosis factor-α (TNF-α) levels 24 h after treatment vs baseline. Studies using human PBMC or isolated monocytes confirmed potentiation of TNF-α production with different TLR agonists, while interleukin (IL)-10 was inhibited
	Gualdoni 2016 [186]		Indoleamine dioxygenase (IDO) activity and neopterin levels					RSV significantly reduced tryptophan levels 2.5 h and 5 h after treatment. The Kyn/Trp-ratio, a measure of IDO activity, was significantly elevated after resveratrol administration
	Pignitter 2016 [187]		Antioxidant capacity in plasma/ reactive oxygen species (ROS) in erythrocytes					Oral administration of RSV did not change total antioxidant capacity, ROS or conjugated fatty acid dienes.

**24**	Gupta 2023 (NCT03253913)[188]	Phase 2, dose-escalating, open-label trial	Change in serum VEGF-D on treatment with RSV and Sirolimus compared with Sirolimus alone. VEGF-D was used as a measure of lymphangioleio-myomatosis cell burden.	Patients with lymphangioleio-myomatosis	RSV given with standard therapy (Sirolimus). Dose escalation study starting at 250 mg once daily for 8 weeks, then 500 mg daily from weeks 8-16, followed by 500 mg twice daily (1g total dose) until 24 weeks.	25 women	18 of the 25 participants (72%) reported at least one AE, with 92 AEs in total and most occurring at the lowest dose of RSV. Most AEs (90%) were categorized as mild, with eight AEs (9%) being moderate and one (1%) being severe. One participant showed worsening of pre-existing gastroesophageal reflux disease that was deemed possibly related to the study drug and necessitated dose reduction. No other dose reductions or interruptions occurred during the study. The most frequently reported AEs were GI in nature, with the most common being GI discomfort, diarrhoea and nausea.	Although the prespecified primary outcome of ≥42% reduction in serum VEGF-D levels after 24 weeks of combined sirolimus and RSV was not observed, there was a statistically significant 10% reduction in mean serum VEGF-D levels during the 24-week study duration. There was an overall improvement in self-reported health status, symptoms, and HRQOL. The combination therapy was considered to be safe and well tolerated.

**25**	Huang 2020[189]	Single blind crossover	Effects on muscle glycogen replenishment and mitochondria biosynthesis	Young male athletes	480 mg RSV or placebo daily for 4 days, crossover design	9, crossover	Not stated	RSV did not improve post-exercise muscle glycogen resynthesis, related glucose uptake or mitochondrial biosynthesis gene expression
	Tsao 2021[190]		Attenuation of oxidative stress, pro-inflammation, and eliminating exercise-induced fatigue					Exhausting time of cycling exercise challenge was not significantly increased in RES vs placebo. IL-6 response was significantly decreased during exercise challenge
**26**	Iannitti 2020 (NCT04258306)[160]	PK study, randomised single blind crossover design	Bioavailability assessment of a new formulation - a solid dispersion of RSV supported by magnesium dihydroxide (Resv@MDH), compared to standard pure RSV	Healthy adult volunteers	Participants were given a solution containing 180 mg RSV from either standard RSV or Resv@MDH (600 mg 30% w/w RSV). After a one-day washout, participants received the other formulation.	6, crossover	Not stated	Resv@MDH displayed a higher C_{max} (6 µM) that occurred earlier (30 min) compared to pure RSV (2 µM at ~90 min), it also had improved bioavailability. The different kinetics suggest a main gastric route for resveratrol absorption from Resv@MDH.
**27**	Iglesias-Aguirre 2022[155]	Metabolism study	To decipher the metabolism of RSV by the human gut microbiota and investigate the possible presence of metabotypes associated with this metabolism	Healthy adult volunteers	150 mg RSV every evening for 7 days	195	No volunteers reported any side effects	Identification of two metabotypes associated with RSV metabolism by the gut microbiota - lunularin producers and non-producers

**28**	Iglesias-Aguirre 2022[191]	PK/metabolism study	To test whether RSV and/or its metabolites are found in the cargo of human plasma extracellular vesicles (EVs), to characterise the encapsulation kinetics in exosome containing EVs (E-EVs), and determine whether RSV intake stimulates EV release.	Healthy adult volunteers	420 mg RSV the evening before attending clinic, and 420 mg on the day of the pharmacokinetic study	16	Zero reported	Of 17 metabolites detected in plasma, 9 were identified in the E-EVs, but not free resveratrol; there was a preference for glucuronide versus sulfate incorporation. The concentration and kinetic profiles of gut microbial metabolites were similar in E-EVs and plasma and RSV intake increased E-EV secretion.
**29**	Joseph 2022 (CTRI/2018/03/012753)[192]	PK study; 2-arm, 4-sequence crossover design	To investigate the bioavailability and PK properties of a resveratrol micelle/hydrogel composite (RF-20) compared to standard unformulated RSV. RF-20 has 20.2 % w/w RSV content.	Healthy volunteers	The study had two phases. In the first phase participants received 80 mg RSV as RF-20 or standard RSV, both in capsule form. In the second phase, participants received sachets of each formulation containing the same dose of RSV which were dissolved in water to make a drink. A	16	Zero reported	RF-20 provided enhanced free resveratrol bioavailability and pharmacokinetic properties compared to the unformulated resveratrol. The enhancement in bioavailability was more when given in sachet form than as a capsule with improved absorption, circulation half-life, and sustained delivery, as compared to the unformulated form.

					minimum washout period of 10 days between the treatments.			
**30**	Kawamura 2020 (UMIN000014836) [150]	Open label dose escalation	Motor function, muscular strength, and creatine kinase (CK) levels	Muscular dystrophy patients	500 mg RSV daily, increased to 1 g at 8 weeks and 1.5 g at 16 weeks then maintained until 24 months.	11	15: Diarrhoea (4x grade 1, 2x grade 2); Abdominal pain (3x grade 1); Upper respiratory infection (1x grade 2, 1x grade 3); Lung infection (1x grade 3); Erythema multiform (1x grade 1); Acne multiform (1x grade 1).<br>No control group as a comparator	Significant increase in mean motor function measure scores from 34.6 to 38.4 after 24 weeks. A two-fold increase was observed in mean Quantitative muscle testing scores of scapula elevation and shoulder abduction. Mean CK levels decreased by 34%

**31**	Kemper 2022 (NCT04668274)[148]	PK study	Characterize the PK profile of JOTROL™, a micellar 10% RSV solubilization formulation in gelcaps, following oral administration of single ascending doses. the effect of food on the PK profile of JOTROL™ safety and tolerability.	Healthy adult volunteers	In part 1, all subjects were sequentially dosed under fasting conditions in an ascending manner across 3 dose levels (200mg, 500mg, and 700mg). A food effect arm was included in part 2 in, which high-fat, high-calorie meals (~800-1000 calories, with 50% of total caloric content derived from fat) were consumed 30 min before administration of JOTROL™ containing 500 mg RSV.	24	A total of 25 treatment emergent adverse events (TEAEs) were reported by 15 out of 24 subjects who received any amount of study drug. 13 events were deemed possibly related. Somnolence was reported by 7 (29.2%) subjects overall. Headache was reported by 5 (20.8%) subjects overall. COVID-19 was reported by 2 (8.3%) subjects overall.	A single 500 mg dose of JOTROL™ generated a C_{max} of 455ng/mL. RSV exposures (AUCs and C_{max}) increased with increasing doses and appeared to be higher than dose-proportional. RSV and its three major conjugates accounted for 40 to 55% of the dose in urine
**32**	la Porte 2010[145]	PK study	PK, Safety	Healthy volunteers	2 g RSV twice daily for 8 days	8	6 AEs reported: Diarrhoea; Transient headache; Evanescent rash. Adverse events were mild and transient. No subject withdrew or was withdrawn	AUC_{12} and C_{max} of t-RES were 3558 ng.h/mL and 1274 ng/mL, respectively, after the standard breakfast. High-fat breakfast significantly decreased AUC_{12} and C_{max} by 45% and 46%, respectively, when compared with the standard breakfast.

							from study or missed a dose because of AEs	Diarrhoea was reported in six of the eight subjects.
**33**	Maia 2012[193]	Open label	Management of endometriosis-related pain measured via pain score in study 1 and COX2 protein expression in study 2	Patients with endometriosis-related pain	30 mg RSV daily for 2 months in both studies	Study 1 single arm trial: 12 on RSV in addition to oral contraceptives. Study 2: 26 on RSV plus standard care; 16 on standard care only.	Not stated	Significant reduction in pain scores. Inhibition of aromatase and COX-2 was significantly greater in eutopic endometrium of patients using combined drospirenone + RSV therapy compared with the endometrium of patients using oral contraceptives alone.
**34**	Mansur 2017 (NCT01668836)[194]	Randomized, parallel, prospective study	Increase in SIRT1 serum concentration and gene expression	Healthy overweight participants	250 mg RSV twice daily for 30 days or a calorie restricted (CR) diet	24 on RSV; 24 on calorie restricted diet of 1000 calories per day	Zero reported	RSV and caloric restriction increased serum concentrations of Sirt1
	Roggerio 2018 [195]		SIRT and RAGE gene expression					RSV and CR increased serum levels of SIRT-1. No change to RAGE serum levels between groups.

**35**	Marchezan 2022[149]	Open label pilot study	Possible beneficial effects of RSV, safety, efficacy, and tolerability, as well as the expression of 8 miRNAs associated with immune modulation	Boys aged 10-13 with Autism Spectrum Disorder	200 mg RSV each morning for 90 days	5	Zero reported	RSV significantly reduced the Aberrant Behaviour Checklist total score and Irritability, with no alteration in Stereotypical Behaviour, Hyperactivity, and Lethargy/Social Withdrawal subscales. On the Clinical Global Impression scale, 3 individuals showed marked improvement in behaviour. RSV treatment significantly increased miR-195-5p, an important modulator of targets related to inflammatory and immunological pathways.
**36**	Marouf 2021 (DISRCTN75392625)[196]	Open-labelled non-controlled clinical trial	Serum level of both type II collagen and aggrecan	Patients with knee osteoarthritis	500 mg daily for 90 days	28	Zero reported	There was a nonsignificant decrease in the serum level of Coll 2-1 and a significant increase in aggrecan serum level after RSV. After 30 days RSV significantly improved the pain score measured by the Visual Analog Scale and Knee injury and Osteoarthritis Outcome Score. Improvements in patients' activity and functional status were also evident at day 30 and kept on for three months.
**37**	Movahed 2020 (IRCT201710108129 N11)[197]	Exploratory pilot study	Safety and efficacy of RSV in Type 1 Diabetes (T1D) patients	Patients with T1D	500 mg RSV twice daily for 2 months; single arm	13	Zero reported	RSV exerted strong antidiabetic and antioxidant effects in patients with T1D

**38**	Nunes 2009[144]	PK, study single arm	PK profile of RSV	Healthy young and elderly adults	200 mg RSV every 8 h for 4 days; one dose taken on the 1st and 4th days	24, single arm	Number not stated; Mild and non-specific; Type not reported	Plasma RSV concentrations following third and eighth doses ranged from 2.5 to 4.0 ng/mL in the young group and 2.1 to 3.1 ng/mL in the elderly group. Steady-state plasma concentrations were attained following 5 or 6 doses of 200 mg
**39**	Ochiai 2019[198]	Cross-sectional study	Pregnancy outcomes in IVF embryo transfer cycles in infertile women	Women undergoing IVF embryo transfer cycles	200 mg RSV once daily for an average of 5.14 ± 5.57 months (SD)	102 on RSV; 2959 controls on standard treatment	Not stated	RSV strongly associated with decreased clinical pregnancy rate [OR, 0.539; 95% CI, 0.341–0.853] and increased risk of miscarriage. However, women on RSV were significantly older with lower serum AMH compared to the controls
**40**	Patel 2010 (NCT00433576)[199]	PK/ biodistribution study	RSV/ metabolite concentrations in blood/colorectal tissue; Anti-proliferative effects of RSV in target tissue.	Colorectal cancer patients	500 mg or 1 g resveratrol daily for 8 days before surgery	10 on 500 mg, 10 on 1 g resveratrol	Zero reported	RSV plus metabolites resveratrol-3-*O*-glucuronide, resveratrol-4'-*O*-glucuronide, resveratrol-3-*O*-sulfate, resveratrol-4'-*O*-sulfate, resveratrol sulfate glucuronide and resveratrol disulfate were identified. RSV and resveratrol-3-*O*-glucuronide were recovered from tissues.

	Patel 2013[200] (reanalysis of samples from NCT00098969 & NCT00433576)		Accurate quantitation of RSV conjugate metabolites in plasma and colorectal tissue	Uses samples from 2 studies - Healthy volunteers and patients undergoing colorectal surgery for colorectal cancer				Accurate determination using metabolite standard curves revealed the average C_{max} for the monoglucuronides, 4'-*O*-sulfate and 3-*O*-sulfates to be ~2.6, 3.8 and 2.9-fold higher, respectively, than previously described. Reanalysis of colorectal concentrations also indicated significant previous underestimation.
**41**	Popat 2013 (NCT00920556)[152]	Phase 2 single arm pilot study	Safety and efficacy of SRT501 resveratrol with bortezomib in patients with relapsed or refractory multiple myeloma	Patients with relapsed or refractory multiple myeloma	5 g micronised RSV (SRT501) daily for 20 days in a 21-day cycle, up to 12 cycles. Some patients also had bortezomib as part of standard treatment	24	15 of 24 patients treated with SRT501 monotherapy were withdrawn from treatment (10 due to AEs, 1 due to AE leading to death and 4 following investigator's decision). All nine patients receiving SRT501 and bortezomib were withdrawn from study (5 due to AEs, 3 following investigators decision and one patient withdrawal). Nausea (79%); Diarrhoea (71%); Vomiting (54%); Fatigue (46%); Anaemia (38%). 54% of patients reported	50% patients had SAEs and two deaths occurred on study (one possibly treatment related; one due to progressive disease). Study demonstrated an unacceptable safety profile, however renal failure was not observed for SRT501 and bortezomib. Renal failure may be specific to MM patients who commonly have renal impairment.

							grade 3 or greater AEs; most common (21%) were haematological (anaemia and thrombocytopenia), 21% renal failure, 13% nausea, 13% infections. 50% had an SAE and two deaths occurred on study (one possibly treatment related; one due to PD).	
**42**	Radko 2013 [201]	PK, bioavailability	Isolation of RSV metabolites from human urine	Healthy volunteers	1 g RSV taken as a single dose	3 on RSV, single group	Not stated	*Trans*-resveratrol-3-*O*-sulfate, *trans*-resveratrol-3,5-*O*-disulfate, *trans*-resveratrol-3,4-*O*-disulfate, *trans*-resveratrol-3-*O*-d-glucuronide, and dihydroresveratrol-3-*O*-d-glucuronide isolated from urine
**43**	Sergides 2016[202]	PK/bio-availability study	Bioavailability and safety	Healthy male and female volunteers	500 mg RSV as a single dose	15 on RSV, single arm	Zero related to study interventions	C_{max} and $AUC_{0\text{-}inf}$ were lower for RSV when compared to values for glucuronidated and sulfated metabolites. RSV 500 mg tablets were well-tolerated by all participants
**44**	Tani 2014[203]	PK study	PK and metabolism of RSV monomer and dimer derivatives from melinjo seed	Healthy volunteers	6.8 mg RSV or 1 g melinjo seed extract on a single occasion	10 on RSV; 5 on melinjo seed extract	Not stated	MSE powder was well-tolerated up to 5000 mg/day. Persistence of RSV monomer and dimer conjugates in plasma may suggest clinical usefulness of MSE powder.

**45**	Theodotou 2017[204]	Randomised trial	Effects of Evelor, a micronized formulation of RSV, in patients with primary hypertension.	Patients with hypertension	10 or 20 mg dapril plus 50 mg RSV daily for 6 months. Control patients received dapril as standard care; No placebo group.	Patients were split into two groups according to degree of hypertension and randomised to dapril with/without RSV. It is not stated how many patients received RSV but group A (Stage I hypertension) had 46 patients, and group B (stage II hypertension) had 51 patients in total with half of each group allocated RSV.	Not stated	Addition of RSV to standard antihypertensive therapy reduces blood pressure to normal levels without requiring additional antihypertensives. Plasma serum glutamate-pyruvate transaminase and gamma-GT were significantly reduced with addition of RSV, indicating that RSV prevents liver damage.
**46**	Theodotou 2019[205]	Pilot study	Liver fat and liver function	Patients with NAFLD	50 mg or 200 mg RSV once daily for 6 months	22 on each dose.	Not stated	Significant reduction in liver fat, serum glutamate pyruvic transaminase, serum glutamic oxaloacetic transaminase and alkaline phosphatase

**47**	Vaz-da-Silva 2008[147]	Open label PK randomized, 2-way crossover study	Whether food effects RSV PK	Healthy subjects	Two consecutive treatment periods separated by 7-day washout. Single-dose of 400 mg RSV following either a standard high fat meal or 8 h of fasting	24 participants, 12 in each treatment sequence	No AEs during fasting period. During the fed treatment period, a total of 7 AEs were reported by 6 (25.0%) subjects. 4 AEs (nausea; toothache; catheter site ecchymosis; dizziness) were reported by 3 (12.5%) subjects, but were considered not related to treatment. 3 AEs (blood electrolytes abnormal, nasopharyngitis and erythematous rash) reported by 3 subjects. AEs were mild, resolved fully and no action was required. There were no SAEs.	The rate of absorption of RSV was significantly delayed by the presence of food, but extent of absorption was not affected.
**48**	Wagemaker 2017[206]	Within-subject controlled study	Influence of RSV-based formulation on inflammatory response in skin.	Healthy volunteers	Application of topical formulation containing 1% RSV or vehicle only, once daily for 7 days	25	Not stated	No significant difference in IL-1Ra/IL-1a ratio from positive control.

**49**	Walle 2004[2]	PK study	Absorption, bioavailability and metabolism of RSV	Healthy volunteers	Single 25 mg dose of oral ^{14}C-RSV and 50 mg of ascorbic acid dissolved in 1 mL of ethanol. Single IV dose of 0.2 mg ^{14}C-RSV.	6 participants, crossover study	Not stated	Absorption of 25 mg oral dose was at least 70%, with peak plasma levels of RSV and metabolites of 491+/- 90 ng/mL) and a plasma half-life of 9.2 +/- 0.6 h.
**50**	Yiu 2015 (NCT01339884)[207]	Open-label, non-randomized, proof-of-principle study	Effect of RSV on peripheral blood mononuclear cell (PBMC) frataxin levels.	Patients with Friedreich Ataxia	0.5 g or 2.5 g RSV twice daily for 12 weeks	13 on 0.5 g; 14 on 2.5 g.<br>No comparator control group	28 on 0.5 g. 64 in group on 2.5g RSV. No SAEs were recorded. Gastrointestinal side-effects were a common, dose-related adverse event. RSV at a dose of 1 g daily was generally well tolerated, apart from one subject who withdrew due to fatigue. The following AEs were reported: upper respiratory tract infection; urinary tract infection; sinusitis; tonsillitis; headache; fatigue; loose stools; diarrhoea; abdominal pain/cramps; nausea; bloating; flatulence; constipation;	PBMC frataxin levels did not change in either dosage group

							dyspepsia; abnormal liver function tests; palpitations; increased creatine kinase level; microalbuminuria; skin rash; lower limb oedema	
**51**	Zhang 2023[208]	PK study	Analysis of urinary resveratrol metabolites	Healthy volunteers	Single oral dose of 600 mg RSV	12	Not stated	Dihydroresveratrol, 3-(4-hydroxyphenyl)-propionic acid, and lunularin were the major microbial metabolites of RSV with interindividual differences.

List of abbreviations used just in this Table and not covered in the main text: AMH, anti-müllerian hormone; COX2, cyclo-oxygenase-2; CYP3A4, cytochrome P450 3A4; FNDC5, Fibronectin type III domain-containing protein 5; GAGS - Global Acne Grading System, IGF-1, insulin-like growth factor-1; IGFBP-3, insulin-like growth factor binding protein-3; IVF, *in vitro* fertilisation; PK, pharmacokinetic; RAGE, receptor for advanced glycation end products; TLR, toll-like receptor; VO_2 max, maximal oxygen consumption.
